# Reasons behind non-adherence of healthcare practitioners to pediatric asthma guidelines in an emergency department in Saudi Arabia

**DOI:** 10.1186/1472-6963-12-226

**Published:** 2012-07-30

**Authors:** Hayfaa A Wahabi, Rasmieh A Alziedan

**Affiliations:** 1 Sheikh Bahamdan Research Chair of Evidence-based Healthcare and Knowledge translation, College of medicine, King Saud University, Riyadh, Kingdom of Saudi Arabia

## Abstract

**Background:**

The prevalence of childhood bronchial asthma in Saudi Arabia has increased in less than a decade from 8% to 23%. Innovations in the management of asthma led to the development of evidence based clinical practice guidelines and protocols to improve the patients’ outcomes. The objectives of this study are to examine the compliance of the healthcare providers in the Pediatrics Emergency Department, in King Khalid University Hospital, with the recommendations of the Pediatrics Asthma Management Protocol (PAMP), and to explore the reasons behind non-adherence.

**Methods:**

This study is designed in 2 parts, a patients’ chart review and a focus group interview. The medical records of all the children who presented to the Pediatric Emergency Department (PED) and were diagnosed as asthmatic, during the period from the 1^st^ of January 2009 to the 31^st^ of March 2009, were reviewed to investigate the compliance of healthcare providers (physicians and nurses) with 8 recommendations of the PAMP which are considered to be frequently encountered evidence-practice gaps, and these are 1) documentation of asthma severity grading by the treating physician and nurse 2) limiting the prescription of Ipratropium for children with severe asthma 3) administration of Salbutamol through an inhaler and a spacer 4) documentation of parental education 5) prescription of systemic corticosteroids to all cases of acute asthma 6) limiting chest x-ray requisition for children with suspected chest infection 7) management of all cases of asthma as outpatients, unless diagnosed as severe or life threatening asthma 8) limiting prescription of antibiotics to children with chest infection. The second part of this study is a focus group interview designed to elicit the reasons behind non- adherence to the recommendations detected by the chart review. Two separate focus group interviews were conducted for 10 physicians and 10 nurses. The focus group interviews were tape-recorded and transcribed verbatim. Theory-based content analysis was used to analyze interviews into themes and sub-themes.

**Results and discussion:**

A total of 657 charts were reviewed. The percentage of adherence by the healthcare providers to the 8 previously mentioned recommendations was established. There was non-adherence to the first 5 of the 8 aforementioned recommendations. Analysis of the focus group interview revealed 3 main themes as reasons behind non-compliance to the 5 recommendations mentioned above and those are 1) factors related to the organization, 2) factors related to the asthma management protocol 3) factors related to healthcare providers.

**Conclusion:**

The organizational barriers and the lack of an implementation strategy for the protocol, in addition to the attitude and beliefs of the healthcare providers, are the main factors behind non-compliance to the PAMP recommendations.

## Background

Bronchial Asthma (BA) is one of the most common chronic diseases worldwide. Epidemiological studies showed high prevalence of asthma in children in many countries all over the globe [1] and the disease is associated with considerable morbidity and a significant economical burden for many countries [2]. In Saudi Arabia asthma is one of the most common chronic diseases, affecting more than 2 million people [3]. The prevalence of childhood BA in Saudi Arabia has increased in less than a decade from 8% to 23% [4,5]. The impact of childhood asthma, as a public health problem for communities and the continuing innovations in its management, led to the development of evidence based clinical practice guidelines and protocols for its management. However, unless these guidelines and protocols are successfully implemented [6], translation of evidence into practice and improvement in patient care will not be achieved [7,8].

Clinical practice guidelines (CPG) are “systematically developed statements to assist practitioners and patient decisions about appropriate healthcare for specific clinical circumstances” [9]. Despite the expected positive impact of the implementation of CPG on the health of the individuals and the quality of medical care by decreasing inappropriate variation in clinical practice, guidelines are not uniformly adopted [10].

Many studies investigated the barriers to the implementation of CPG in healthcare and the effective strategies for translating research into practice, however it is recognized that identification of local barriers to change is pivotal to changing practitioners’ behavior towards adoption of guidelines [11].

In this study we examine the barriers to the adoption of the asthma clinical practice guideline by the healthcare professionals in the pediatrics emergency department (PED), focusing on the guidelines as an innovation and employing the theory of diffusion of innovation to investigate the barriers to implementation.

Diffusion of innovation, as introduced by Rogers et al, is the process by which a new practice (an innovation) is communicated over time among members of a social system [12]. The process of adoption of innovation develops in 4 stages which are: knowledge or awareness, persuasion, decision and implementation. The concept was further developed by Greenhalgh and her colleagues to include innovation in health services organizations [13]. They suggested that diffusion of innovation is influenced by factors related to the attribute of the innovation, the adopters’ characteristics, the context or the environment where the innovation is implemented and the dissemination efforts [13].

In this study we considered the Pediatric Asthma Management Protocol (PAMP) as the innovations, the healthcare professionals working in the emergency department as adopters and the organization (department and the hospital) is the context or the environment in which the PAMP is implemented. We investigated the barriers for implementations through the lens of the factors which influence innovation adoption, including the characteristics (attributes) of the PAMP, the characteristics of the healthcare professionals (adopters’ characteristics) and the environmental factors (context) which constitutes the complex environment of the department and the hospital as well as the patient as a stakeholder.

## Method

The protocol for this study was approved by King Saud University Institutional Review Board and by the PED.

This study is designed in 2 parts; the first part is a patients’ chart review to investigate the compliance of healthcare providers (physicians and nurses) to 8 recommendations of the PAMP and the second part is a focus group interview to elicit the reasons behind non- adherence to the recommendation detected by the chart review. The 8 key recommendations which were deduced from the literature [14] were considered as indicators for evidence based best practice.

King Khalid University Hospital (KKUH) in Riyadh, one of King Saud University Hospitals, is a tertiary referral center. The hospital has 750 beds and provides services for all major specialties as well as subspecialties such as infertility and plastic surgery. The hospital provides emergency services through the adult and pediatric emergency departments.

During the last 2 years KKUH has witnessed considerable expansion and reform to face the increase in the catchment area covered by its services and to fulfill the requirements for the accreditation.

To unify the quality of healthcare delivery, the hospital accreditation requires implementation of clinical guidelines in all departments with high turnover of patients such as the emergency department. Sheikh Bahamdan Research Chair of Evidence Based Healthcare and Knowledge Translation is commissioned by the Quality Department to outline strategies for effective implementation of Clinical Practice Guidelines (CPG) in high demand areas. The PED is one of the busiest departments in the hospital; there were 42,635 visits to the department in 2009, 5% of which were for BA.

The PED has few protocols to unify the management of certain conditions which are seen frequently in the PED including a protocol for management of acute BA.

The PAMP is based on the recommendations of the Saudi Initiative for Asthma (SINA), which is a national guideline for the management of asthma in adults and children [3]. SINA was adapted from Global Initiative for Asthma (GINA) and the National Asthma Education and Prevention Program [3,15].

The PAMP was developed by the members of the pulmonology department in KKUH without active contribution from the staff in the PED. The protocol has no reference to the parent guidelines from which it was developed. Implementation of the protocol started in 2005.

The PAMP was introduced to the PED staff during one of the department monthly meetings, which is usually attended by the physicians and the head nurse. Following the meeting the protocol was approved for implementation. The PAMP was not accompanied by an implementation strategy.

### Chart review

To investigate the healthcare providers’ adherence to the existing PAMP; the file numbers of the patients seen by the PED with the diagnosis of BA during the period from the 1^st^ of January 2009 to the 31^st^ of March 2009, were extracted from the PED admission registry book, then the patients charts were retrieved for data collection.

Data were extracted from the medical record using a pre-formatted data collection sheet. For the purpose of establishing the percentage of patients with mild and moderate asthma who received Ipratropium, and due to infrequent documentation of asthma severity grading in patients’ records, a retrospective grading was done by the two authors at the time of data collection using the same grading score of the PAMP and the vital signs recorded for each patient which were available for all patients included in this study.

Any feature of moderate asthma led to classification as moderate and any feature of severe asthma led to the classification as severe.

Data collected included the number of healthcare providers complying with the following 8 recommendations which were considered as frequently encountered evidence-practice gaps in the management of acute BA in children [14]:

1. Documentation of asthma severity grading by the treating physician and nurse as either mild, moderate or severe.

2. Administration of Salbutamol using an inhaler via a spacer.

3. Prescription of systemic corticosteroids to all cases of acute BA.

4. Documentation of parental education for the home asthma management plan.

5. Management of all cases of asthma as outpatients unless diagnosed as severe or life threatening asthma.

6. Prescription of Ipratropium for children with severe asthma only.

7. Prescription of antibiotics for children with evidence of chest infection only.

8. Chest x-ray requisition for children with signs of chest infection only.

The cut-off value for each recommendation was extracted from the PAMP recommendations; when the recommendation includes (all patients) we considered the cut-off value to be 100% of patients and this applied to recommendations 1–4. For recommendations 5 and 6 we performed retrospective asthma grading of all patients included in the chart review and we considered the cut-off value as the percentage of children who presented with severe or life threatening asthma. For recommendations 7 and 8 we calculated the cut-off value from the percentage of children with asthma who were febrile when they presented to PED. Cut-off values were rounded to the nearest 10.

The percentage of healthcare providers adhering to each one of the 8 recommendations aforementioned was calculated.

Following the analysis of the focus group interviews, further analysis of the chart review data was performed to explore the adherence of the healthcare providers to the PAMP recommendations stratified by patients’ age group (≤ 3 and >3 years). The analysis was performed to substantiate or refute the claims of the healthcare providers about the difficulties to apply the PAMP recommendations on young children as the reason for non-adherence. Furthermore the charts of the children who re-visited PED within a week of the first visit with asthma were identified from admission registry book and were excluded from the final analysis.

Other data collected included the gender and the age of the child.

### Focus group interview

The first part of this study showed that there were 5 recommendations of the PAMP which were not adhered to by the healthcare providers namely; grading of asthma severity, the prescription of Salbutamol nebulizer instead of the inhaler, over-prescription of Ipratropium for mild or moderate asthma, under-prescription of corticosteroids and lack of documentation of parents’ counseling for home treatment plan (Table 1).

**Table 1:** Recommendations of the PAMP inadequately adhered to by the healthcare providers

**Recommendation**	**Cut-off point**	**All Study Groups n = 657(%)**	**Group ≤3 years n = 328 (%)**	**Group > 3 years n = 329 (%)**
Documentation of Asthma severity grading	100%	3 (0.4)	1 (0.3)	2 (0.6)
Salbutamol metered-dose inhaler use	100%	0.0	0.0	0.0
Ipratropium prescription	≤ 10%	316 (48.1)	149 (45.4)	167 (50.7)
Corticosteroid prescription	100%	188 (28.6)	83 (25.3)	105 (32.0)
Documented parents’ education	100%	2 (0.3)	1 (0.3)	1 (0.3)

PAMP = Pediatric Asthma Management Protocol.

We conducted focus group interviews to explore the opinions of the healthcare professional about the reasons behind non-adherence to the aforementioned recommendations of the PAMP. The focus group interview approach was chosen to facilitate fuller expression of the participants’ opinions, capitalizing on the group dynamic and interaction during the discussion [16,17].

At the time of data collection for this study, the PED was staffed by 12 physicians who covered 8 hour shifts, and 20 nurses who covered 12 hour shifts. There were 4 consultant physicians, 6 specialists and 2 residents. All physicians are bilingual; speaking both Arabic and English except for 2 who speak English and Urdu. The nursing staff includes one head nurse, 10 senior and 10 junior nurses. All nurses speak Filipino as their first language and English as the language of communication with the other professionals in the department. On joining the department, all nurses were given courses in Arabic language to help communication with the patients.

We used a purposive sampling to recruit nurses and physicians for the focus group. The participants of the focus group were staff who hold substantive posts (locum staff excluded) and who have spent a year or more in the PED. All nurses and physicians working in the PED were invited to participate in the focus group interview; however participation was totally voluntary with the option to withdraw at any time during the interview.

At the start of each focus group the study objectives were explained to the participants, confidentiality was granted and the permission of the participants to tape-record the session was obtained.

During November and December 2009; 2 separate focus group interviews were conducted for 10 physicians and 10 nurses by the authors (RZ, HW), at the PED during a time convenient to the participants.

The questions for the semi-structured interview for focus group, which were developed by the authors, were asked to the participants to start the focus group discussion (Additional file 1).

The participants were encouraged to discuss the barriers for implementation of the 5 recommendations of the PAMP which were not adhered to, quite freely and only when the discussion was out of track, did one of the authors interfere to direct the discussion back to the study topic [16]. Focus group interviews were conducted in English language (the language for reporting medical findings). At rare occasions Arabic speaking physicians spoke in their first language; at those times one of the facilitators encouraged them to express themselves in English language so as not to disturb the group dynamic for English speaking physicians. At all times one of the two facilitators (RZ and HW) kept field notes while the other facilitator ensured the coverage of all the topics on the topic guide.

To deepen our understanding of the issues raised during the focus group as reasons behind non-compliance to the recommendations, the two authors performed 4 additional individual semi- structured interviews, one with each of the following key personnel; the head of department, the head nurse, the pharmacist responsible for the equipment and drug supply to the PED and the most senior physician in the department. The head of the department and the pharmacist did not attend the large focus group interview while the senior nurse and the senior physician did. The purpose of the interviews was to further explore the reasons raised by the focus groups to be behind non-adherence to the PAMP.

### Focus group data analysis

The interviews were tape-recorded, transcribed verbatim and independently checked for accuracy by the two authors. Transcribed interviews were read and text which appears to describe a barrier to the implementation of the asthma protocol was highlighted. Subsequently all the highlighted text was coded using the predetermined themes applying a directed qualitative content analysis as described by Hsieh et al. [18]. For this method of content analysis and to determine the main themes of the study, we draw on the conceptual model of the innovation diffusion theory [12] and its application in healthcare organizations [13]. The following themes were derived from the theory; perceived characteristics of the innovation (PAMP), characteristics of the adopters (healthcare professionals), and the context (organizational factor). The sub-themes emerged from the interviews. According to the codes, quotes were sorted into themes and sub-themes. Analysis was conducted by the two authors and disagreement was resolved by discussion.

## Result

### Chart Review

A total of 817 charts were reviewed, after exclusion of the children who attended the PED as revisit, 657 charts were analyzed. 328 (49.9%) of the children were 0–3 year old while children older than 3 years accounted for 329 (50.1%) of the study population (Table 2). Retrospective grading of asthma revealed that 339 (51.6%) of the cases were mild asthma, while severe asthma amounts for only 53 (8.0%) of the cases seen during the study period (Table 3). The children documented to be febrile at presentation and were eligible to receive antibiotics and for whom a chest x ray should be requested were 98 (15%). The percentage of adherence by the healthcare providers to the 8 previously mentioned recommendations was established. In 5 recommendations there was either no adherence or the adherence was suboptimal for the total study population as well as for both sub-groups of children (≤ 3 and >3 years) (Table 1), while adherence was adequate to the 3 remaining recommendations (Table 4).

**Table 2:** Age range of the patients who visited the PED with the diagnosis of asthma during the study period (n = 657)

**Patient age range (Year)**	**n (%)**
**0-3**	328 (49.9)
**4-6**	119 (18.1)
**7-9**	105 (16)
**10-12**	105 (16)

PED = Pediatric Emergency Department.

**Table 3:** Retrospective grading of asthma in the study population

**Asthma severity**	**Percentage from the total studied population n (%)**
Mild	339 (51.6)
Moderate	265 (40. 4)
Severe	53 (8.0)

**Table 4:** Recommendations of the PAMP adequately adhered to by the healthcare providers

**Recommendation**	**Cut –off point**	**All Age Groups n = 657 (%)**	**Group ≤ 3 years n = 328(%)**	**Group > 3 years n = 329 (%)**
Antibiotics prescription	≤ 10 %	46 (7.0)	25 (7.6 )	21 (6.4)
Admission to hospital	≤ 10 %	14 (2.1)	5 (1.5 )	9 (2.7)
Chest x-ray requisition	≤ 15 %	72 (10.9)	34 (10.3)	38 (11.5)

PAMP = Pediatric Asthma Management Protocol.

### Focus group

The age range of the interviewees was 25-54 years. Each interview lasted approximately 45–60 minutes.

From the analysis, 3 main themes were recognized as reasons behind non-compliance to the 5 recommendations mentioned above and those are 1) factors related to the PAMP 2) factors related to healthcare providers 3) factors related to the organization. Themes and sub-themes are shown on Table 5.

**Table 5:** Themes and sub-themes of the focus group

**Main themes**	**Sub-themes**
**Factors related to the PAMP**	· Lack of clear development and dissemination plan of the protocol
	· Lack of implementation strategy
	· Lack of recommendations suitable for the characteristics of some patients seen in PED
**Factors related to healthcare providers**	· Language barrier between nurses and parents.
	· Lack of awareness of the protocol
	· Lack of familiarity with the recommendation of the protocol.
	· Disagreement with recommendation
**Factors related to the organization**	· Staff and bed shortage
	· Deficient outpatient referral system
	· Availability and cost of certain devices

PAMP = Pediatric Asthma Management Protocol. PED = Pediatric Emergency Department.

Physicians and nurses had similar opinions about the barriers to the implementation of the 5 aforementioned recommendations. Themes and sub-themes are detailed below.

### Factors related to the PAMP (The perceived innovation attributes)

#### Lack of clear development and dissemination plan for the PAMP

The staff in the PED was not directly involved in the development of the PAMP and because there is no reference to the parent guideline from which the protocol was developed; it was discredited by many doctors and nurses. The protocol was introduced to the department once during the monthly departmental meeting, which was attended by the medical staff who were not on duty at that time, in addition to the head nurse. Thus many physicians and nurses were not aware of the existence or the content of the protocol or their role in following its recommendations.

Physician: the bronchial asthma protocol…..I am not quite sure where it came from…Most of us are following the North American guidelines…I am not sure if this is adapted from their guidelines.

Physician: I am not sure if we have a written protocol for the treatment of asthma…I think there was a plan to do that …but a finished protocol I don't think so.

Nurse: These guidelines are for doctors…I don’t know much about them and doctor should stay up-to-date and update the clinical guidelines and our role as nurses to follow the order.

#### Lack of implementation strategy

The recommendations of the PAMP were not accompanied by an implementation strategy such as; a written instruction for the parents on discharge of the child from the PED (action plan) or a user friendly worksheet for easy documentation of asthma severity grading and parents’ education. Frequently asthma grading and parental education is done but not documented.

Physician: okay ….grading of asthma we all do it, but they have to add space for grading to the admission sheet like in other hospitals, so I remember to write it down …..

Nurse: we can all follow the protocol if they add a worksheet to patient’s chart and it has to be simple just to put a tick…you know we are very busy here.

Physician: I worked in other hospitals and they have a written action plan for the parents on discharge…It can spare us a lot of time we spend on explaining to the parents …I mean most of these parents can read….

#### Lack of recommendations suitable for the characteristics of patients seen in PED

A considerable number of the children who visited the PED with asthma were 0–3 years old (Table 2) and all physicians stated that grading and diagnosing asthma in such young age group is very difficult especially when the child is crying so they consider them as severe asthma and treat them as so. Unlike many published guidelines [15], the PAMP has no special section on guidance of how to manage children ≤ 3 years of age.

Physician: yes we used Ipratropium in 99% of the children who have moderate to severe asthma, because most of the cases are less than 3 years…..it’s difficult to get the signs while they are crying and again most of these kids progress very quickly to severe asthma if not treated promptly.

However the non-adherence of the healthcare providers to the PAMP recommendations was evident even in children > 3 years of age (Table 1).

### Factors related to healthcare providers (adopters)

#### Language barrier between nurses and parents

Communication between parents and nurses is affected by the language barrier because most of nurses in PED are non-Arabic speakers.

Nurses: you see… we can’t explain to the parents in details the use of inhalers and spacers ….we had Arabic courses and stuff but it’s not enough to explain that……see sometimes we get a mother who speaks English but this is rare.

#### Lack of awareness of and familiarity with the PAMP

Some of the physicians and nurses are not aware that there is a protocol for asthma management, others are not familiar with the recommendations of the protocol and they did not think it is different from their practice.

Physician: I am not quite aware that there is a protocol for asthma management here….anyway management of asthma is all the same protocol or no protocol.

Nurse: I am not sure about these protocols…they are made for the doctors not us.

#### Disagreement with recommendation

Physicians disagree with some recommendations of the protocol and believe their clinical judgment should supersede the recommendation in certain circumstances.

Physician: I don’t think all mild asthma cases requires steroids, some of these kids respond beautifully to one dose of Salbutamol and they are ready for discharge….I am not giving them steroids because they don’t need it….

### Factors related to the organization (The context)

#### Staff and bed shortage

Interviewed physicians and nurses stated that high number of patients during winter and the staff shortage were the reasons behind non-compliance to more than one of the recommendations of the PAMP. They stated that lack of parental education is mainly due to the busy PED and staff shortage including the lack of a health educator.

The staff shortage is another reason that Salbutamol is given through a nebulizer rather than an inhaler because one nurse can follow more than one patient treated with nebulizer at one time; however using the inhaler will require “one to one care”.

Bed shortage in the PED is one reason that Ipratropium is used in mild or moderate asthma to synergize with the action of the Salbutamol and ensure quicker response and discharge of the patients from PED for the other waiting patients to be seen.

We could not confirm that adherence to PAMP recommendations was subjected to seasonal variation because the chart review data was collected between January and March (winter) rather than all through the year (e.g. interrupted time series);

Nurse: In winter, we see a lot of asthmatic patients, and we are very busy attending to 10 sometimes 20 patients at a time and we can’t afford educating all these people, I mean we have shortage of staff and like….in other places there is special staff to do this…..

Physician: I know there are no spacers to use with the inhalers now, but even when they were available it was not possible to use them especially in winter when there is big rush of patients the nurse can follow up more than one child with the nebulizer but not when the medication is given by the inhaler.

Physician: I believe Ipratropium saves the day when there are 20 or more waiting to be seen and all the 9 beds are full, it gives quicker action with Salbutamol and you can discharge the child quicker to be able to see the other waiting children…..

#### Deficient outpatient referral system

In the absence of a recognizable system for referral of patients to outpatient services for continuing care of asthma, patients frequently visit the emergency departments for follow up management rather than for emergency treatment.

Physician: The main problem of this department is the revisit, not for a new attack but either to have medicine or just to be reassured that the child is OK.

I won’t give steroids to a child who has just finished the last dose a day ago or still taking it …It is all because they have no outpatient to go to….If you look closely you will find that all of those who did not receive steroids are revisiting…..they’re already on steroids…

Based on the previous quote we excluded children who attended the PED as revisit from the analysis; however the non-adherence of the healthcare providers to the PAMP recommendations was evident in patients who presented acute asthma (Table 1).

#### Availability and cost of certain devices

Availability and the cost of certain devices, such as spacer, affect the adherence to the recommendation of using a metered dose inhaler rather than the nebulizer to deliver the medication.

Nurse: spacer ….we can’t use Salbutamol inhaler in the PED instead of nebulizer because spacer isn’t in stock in the pharmacy for a long time……

Physician: do you know how much the spacer cost? It cost 70 Riyals while the nebulizer is for 4 Riyals only. I don’t think the head of the department can justify the cost difference if he asks for inhalers instead of nebulizers in the PED.

## Discussion

Protocols are tools for translating evidence into practice; they are intended to integrate evidence based guidelines into healthcare provision and to improve the quality of patient care. Nevertheless implementation of evidence into practice is not a linear or passive process and that it entails both organizational and individual behavioral changes [19]. Addressing barriers to implementation of evidence based guidelines is a pivotal process which is integrated in many knowledge translation models [20-22]

In this study the 3 themes suggested in the theoretical framework covered all the perceived barriers expressed by healthcare professionals in the PED, which shows that the framework is suitable for the evaluation of implementation of guidelines as an innovation (Table 5).

Many of the perceived attributes of the protocol impeded its adoption by the healthcare professionals, such as the lack of participation of the target group of users (physicians, nurses and patients) in the development of the protocol, which is a known barrier for implementation of guidance [23], and the fact that the original guidelines from which the protocol was developed was not known, affecting its credibility by some of the interviewed physicians.

Developing the protocol and introducing it to the department in the general meeting was the only dissemination effort made by the department to translate asthma guidelines into practice. Simplifying the recommendations of the guidelines into a format of a protocol, or a clinical pathway, overcomes a known barrier for implementation of evidence and wit, the complexity of some guidelines [23]. However, a single implementation strategy may not be as effective as a multifaceted approach to ensure the awareness of the healthcare professionals of the existence of the guideline, to increase their familiarity with its recommendations and to detect and address barriers to the implementation of these recommendations [24,25]. Some of the PAMP recommendations were perceived by the healthcare professionals not to be applicable to a large number of the patients attending the PED. Nearly 50% of the patients who attended the PED during the study period were 0–3 years old (Table 2). According to most of the interviewed physicians, applying some of the recommendations of the PAMP such as staging of asthma severity is not easy in this age group. Patients’ characteristics, such as co-morbidity, was found by other investigators to affect the adoption of guidelines by healthcare professionals [26]. Providing guidance for a special group of people defined by co-morbidity or physiological characteristics is suggested to provide evidence based management for these special groups of patients [27]. Moreover a number of guideline developers are providing special guidance for patients with certain physiological characteristics such as pregnancy as well as for specific age groups [28]. However in this study we did not find evidence from the quantitative data to support the claims that adherence to the asthma protocol is influenced by the patient age (Table 1).

The characteristics of the healthcare professionals in this study were found to influence the adoption of guideline recommendations. Lack of awareness of the existence of the protocol, which represents the lack of knowledge about the innovation, an essential first step in Rogers et al theoretical frame for the innovation diffusion process [12], and the disagreement with some of its recommendations, such as the use of corticosteroids for mild asthma were factors behind non- adherence of some professionals to the recommendations. Similar characteristics of healthcare professionals were found by other studies [26,29]. However a unique finding in this study is the language barrier between the nurses and the patient’s parents due to the fact that Arabic is not the first language for most of the nurses in the Saudi health settings.

Organizational or contextual barriers such as limited time and personnel in addition to the lack of some equipment and devices were raised repeatedly by the focus group and were considered among the most important barriers for implementation of the asthma protocol. Similar barriers were recognized by other investigators [26,30]. A unique finding to this study is the impact of the defective system for patients’ referral to outpatient services, thereby increasing the load on the emergency department by increasing the revisit rate and affecting the continuity of care for the patients. Almost 20% of the patients who presented to the PED and were diagnosed as asthmatic were not in the acute stage of the disease; nevertheless they will create confusion about the consistent use of the PAMP in the department and difficulty in the interpretation of any audits designed to evaluate implementation.

This study reflects the efforts of exploring the barriers to the implementation of good medical practice by applying a theoretical framework to establish a tailored implementation strategy in the future.

The results of the study has many implications to practice in the PED setting and in similar setting in Saudi Arabia including the importance of designing an effective dissemination and implementation strategy for clinical guidelines and protocols, in addition effective policies should be implemented to improve communication between medical and nursing staff and the patients or their parents including addressing language barriers. Since the introduction of the PAMP in 2005, the department did not conduct an audit to assess the success of implementation, which might have contributed to the current state of non-adherence to most of the recommendations. Regular audit and redesign of the strategies of implementation should be an integral part of any future plans for evidence translation.

The strength of this study comes from the iterative employment of both the quantitative and the qualitative design to explore the barriers to the adherence to the asthma guidance in PED.

We are aware of the limitations of this study including that it investigated barriers to evidence-based clinical practice without linkage to the patients’ outcome, such as the number of revisits. However parental education, which one of the main factors for improving revisit rate, was provided to only 2 patients, which invalidate the value of linking outcome to adherence. It is worth noting that this study investigated the adherence of the healthcare professionals to the PAMP recommendations during winter (3 months), when most of the BA patients present; however the results might not reflect the adherence during the rest of the year when the demand for the service is not as high. Another limitation is that the focus group did not include all the staff working in the PED and it may have missed some important opinions.

## Conclusion

This study provided a comprehensive investigation of the barriers impeding the implementation of the PAMP in the PED from the perspective of the healthcare professionals working in the department. The organizational barriers and the lack of an implementation strategy for the protocol, in addition to the attitude and beliefs of the healthcare providers, are the main factors behind non-compliance to the PAMP recommendations. The study provides the substrate for a tailored strategy for PAMP implementation.

## Competing interest

The authors declare that they have no competing interests.

## Authors’ contributions

HW and RZ conceived the idea of the research; RZ collected data for the quantitative part of the study and HW and RZ conducted and analyzed the focus group interviews, HW written the manuscript and RZ reviewed it.

## Pre-publication history

The pre-publication history for this paper can be accessed here:

http://www.biomedcentral.com/1472-6963/12/226/prepub

## Supplementary Material

Additional file 1 Questions for the Semi-structured Focus Group Interview. Click here for file
