# Supplementary material for: Reasons behind non-adherence of healthcare practitioners to pediatric asthma guidelines in an emergency department in Saudi Arabia
Source: BMC Health Serv Res. 2012 Jul 30;12:226. doi: 10.1186/1472-6963-12-226 (PMC3464177; doi:10.1186/1472-6963-12-226)
Supplement: Additional file 1 — Questions for the Semi-structured Focus Group Interview. [file 1472-6963-12-226-S1.doc]

**Appendix 1**

Questions for the Semi-structured Focus Group Interview

| **Topic** | **Specific focus group question** |
| --- | --- |
| The PAMP protocol in general | What are the barriers which might prevent the adherence to the PAMP in your opinion? |
| Documentation of asthma grading | The PAMP in the PED recommended that asthma grading (mild, moderate or severe) is documented on the patient’s records on admission, why, in your opinion, a physician or a nurse would not follow this recommendation? |
| Use of salbutamol inhaler instead of nebulizer | The PAMP in the PED recommended that an inhaler should be used for delivery of salbutamol rather than a nebulizer, why, in your opinion, a physician would not follow this recommendation? |
| Ipratropium should be prescribed for severe asthma only | The PAMP in the PED did not recommend the use of Ipratropium for mild or moderate asthma, why, in your opinion, a physician would not follow this recommendation? |
| Prescription of corticosteroids for all patients presenting to PED with asthma | The PAMP in the PED recommended the prescription of corticosteroids for all patients presenting with asthma. Why, in your opinion, a physician would not follow this recommendation? |
| Documentation of parents education on home treatment | The PAMP in the PED recommended that on discharge of the patients from the PED, the parents should receive a written and verbal education about when to bring the child back to the PED and about how to use the home medication for asthma and this should be documented on the patient’s record, why, in your opinion, a physician or a nurse would not follow this recommendation? |

PAMP= Pediatric asthma management protocol. PED=Pediatric emergency department
